# Rapid on-site detection of *Pasteurella multocida* in cattle using an LFS-based RPA-CRISPR/Cas12a assay

**DOI:** 10.1186/s13620-026-00360-4

**Published:** 2026-09-07

**Authors:** Xueshu Han, Ruiguo Wang, Yang Ni, Zhenjun Sun, Yuping Jin, Zicheng Xing, Yuqing Guo, Haonan Wang, Shaochun Liu, Yalin Diao, Huanqi Liu, Liang Chi

**Affiliations:** College of Veterinary Medicine, Qingdao Agricultural University, Qingdao, 266109 China

**Keywords:** Bovine respiratory disease (BRD), CRISPR/Cas12a, Lateral flow strip, On-site rapid detection, *Pasteurella multocida*

## Abstract

**Background:**

*Pasteurella multocida* is an important bacterial pathogen associated with bovine respiratory disease (BRD), causing substantial economic losses in cattle production worldwide. Rapid field detection of *P. multocida* remains challenging because conventional diagnostic methods are often time-consuming and require specialized laboratory equipment. This study aimed to establish a rapid visual assay for *P. multocida* to support field diagnosis and disease management in cattle.

**Methods:**

A recombinase polymerase amplification (RPA)-CRISPR/Cas12a assay targeting the species-specific *kmt1* gene of *P. multocida* was developed and combined with lateral flow strips (LFS) for instrument-free visual detection. The assay performance was comprehensively evaluated based on sensitivity, specificity, repeatability, and validation using clinical samples.

**Results:**

Under optimized conditions, the RPA amplification was completed within 15 min at 37 °C, and the CRISPR/Cas12a reaction generated clear visual signals on the LFS. The assay achieved a detection limit of 10^1^ copies/µL, showing higher sensitivity compared with conventional PCR. No cross-reactivity was observed with ten common bovine pathogens, indicating high specificity. Repeatability testing demonstrated stable and consistent performance. Among 40 clinical bovine samples, the assay results were fully consistent with conventional PCR, successfully identifying 17 positive and 23 negative samples.

**Conclusions:**

The established LFS-based RPA-CRISPR/Cas12a assay enables rapid, sensitive, and instrument-independent detection of *P. multocida*. This method provides a practical point-of-care testing (POCT) tool for rapid on-site screening of *P. multocida* in resource-limited veterinary settings.

**Supplementary Information:**

The online version contains supplementary material available at https://doi.org/10.1186/s13620-026-00360-4.

## Introduction

*Pasteurella multocida* is a major bacterial pathogen associated with BRD, causing substantial economic losses in the cattle industry worldwide [1]. The bacterium mainly colonizes the respiratory tract of cattle and can lead to severe respiratory symptoms, pneumonia, and hemorrhagic septicemia under conditions of stress or immunosuppression [2, 3]. In addition, *P. multocida* frequently co-infects with other respiratory pathogens, such as *Mannheimia haemolytica* and *Mycoplasma bovis*, further aggravating disease severity and increasing mortality [4, 5]. Therefore, rapid and accurate detection of *P. multocida* is essential for timely disease control and herd health management.

Traditional diagnostic methods for *P. multocida* primarily rely on bacterial isolation, serological assays, and molecular detection techniques [4, 6, 7]. However, bacterial culture is labor-intensive and time-consuming, while serological assays often suffer from limited sensitivity and specificity [7]. Although polymerase chain reaction (PCR)-based methods provide improved sensitivity, they generally require specialized laboratory equipment and trained personnel, limiting their applicability in field and resource-limited veterinary settings [8]. Consequently, there is an urgent need for rapid, sensitive, and field-deployable diagnostic methods for on-site detection of *P. multocida*.

RPA is an isothermal amplification technique capable of rapidly amplifying nucleic acids under mild temperature conditions (37–42 °C) without the need for thermal cycling equipment [9, 10]. Recently, CRISPR/Cas-based detection systems have attracted increased attention in molecular diagnostics owing to their high specificity and rapid detection capability [11–13]. Guided by a specific CRISPR RNA (crRNA), the Cas12a endonuclease precisely recognizes target DNA sequences and subsequently triggers the trans-cleavage of single-stranded DNA (ssDNA) reporters, enabling visual or fluorescent signal generation [13, 14]. When coupled with RPA, this system integrates rapid isothermal amplification with highly specific nucleic acid recognition, substantially improving detection sensitivity and accuracy under simple operating conditions. Consequently, RPA-CRISPR/Cas12a-based detection platforms have been widely implemented in pathogen diagnosis, including foodborne pathogens, human infectious diseases, and veterinary diagnostics [15–17].

Previous studies have reported fluorescence-based RPA-CRISPR/Cas12a assays for the detection of *P. multocida* [18]. However, fluorescence-based detection still depends on specialized fluorescence readers or laboratory instruments, which limits its applicability for rapid field diagnosis in veterinary practice. In contrast, lateral flow strip (LFS)-based visualization provides a user-friendly and instrument-independent readout, rendering the assay more suitable for point-of-care testing (POCT) and field deployment. To ensure high diagnostic accuracy in diverse clinical isolates, selecting a robust molecular marker is essential; the species-specific *kmt1* gene represents a well-established and highly conserved genomic target widely used for *P. multocida* identification [19]. Therefore, the present study aims to develop and evaluate an LFS-based RPA-CRISPR/Cas12a assay targeting the species-specific *kmt1* gene of *P. multocida*. The assay performance was systematically assessed in terms of sensitivity, specificity, repeatability, and clinical sample validation to explore its potential for the rapid, on-site diagnosis of bovine *P. multocida* infection.

## Materials and methods

### Plasmid construction

The species-specific *kmt1* gene (GenBank accession number KX348143.1) was amplified using genomic DNA of the standard *P. multocida* strain as the template. To achieve directional assembly, PCR primers were designed using Primer Premier 5.0 to append specific 15–20 bp homology arms complementary to the terminal sequences of the linearized vector onto the 5′ ends of the target fragment (Supplementary Table S3). The primers were synthesized by Tsingke Biotechnology (Beijing, China). Following agarose gel electrophoresis and purification, the PCR amplicons were seamlessly cloned into the pre-linearized pUC57EVL vector (pUC57EVL Seamless Cloning Kit; Biomed, Beijing, China) and transformed into *Escherichia coli* DH5α competent cells (Hunan Accurate Biological Engineering Co., Ltd., China). Single colonies were selected and cultured for plasmid DNA extraction. The recombinant plasmids were subsequently verified by Sanger sequencing to ensure both sequence accuracy and the correct insertion orientation. Plasmids with sequences fully matching the reference sequence were chosen as standard templates, aliquoted, and stored at − 80 °C for further use.

### Development and optimization of the RPA reaction system

Five pairs of RPA primers, designed based on the *kmt1* gene sequence (GenBank: KX348143.1), were created using Primer Premier 5.0 software and synthesized by Tsingke Biotechnology Co., Ltd (Beijing, China). The sequences of the primer pairs are shown in Table 1.

**Table 1:** Primer sequences for RPA amplification

Name	Sequences(5’→3’)	Length
*kmt1*-F1	ACCGATTGCCGCGAAATTGAGTTTTATGCC	421 bp
*kmt1*-R1	ACTGACACGATCAAACCGTTGAACACGAAG	
*kmt1*-F2	TTGTGAGTGGGCTTGTCGGTAGTCTTTTATTTGGC	396 bp
*kmt1*-R2	CCGCTCTGTCGTTAATGGCTTCAATAATGGCCATA	
*kmt1*-F3	CCAATCTGCTTCCTTGACAACGGCGCAACTGATT	250 bp
*kmt1*-R3	ACTGACACGATCAAACCGTTGAACACGAAGAA	
*kmt1*-F4	AGCCAATCTGCTTCCTTGACAACGGCGCAAC	205 bp
*kmt1*-R4	AATTAAACCGCTCTGTCGTTAATGGCTTCAA	
*kmt1*-F5	TTGTCGGTAGTCTTTTATTTGGCTTGTGGCAA	376 bp
*kmt1*-R5	AACGAACTCGCCACTTTTTGTTTCATTTGGA	

The RPA reaction was performed in a 20 µL reaction system using a commercial RPA kit (Shenzhen EZassay Biotechnology Co., Ltd., China) according to the manufacturer’s protocol, with the integration of Uracil-DNA Glycosylase (UDG) system to prevent aerosol carry-over contamination. Briefly, the extracted *P. multocida* genomic DNA template was first pre-treated by incubating it with 10× UDG at a 9:1 volume ratio for a short period. Next, a master mix containing 10 µL of 2× Rehydration Buffer, 1 µL of forward RPA primer (10 µM), 1 µL of reverse RPA primer (10 µM), and RNase-free water was prepared to resuspend one tube of lyophilized RPA enzyme pellet. After adding the UDG-pretreated DNA template, 2 µL of starter was added to the mixture. Finally, 1 µL of 1× UDG was added to complete the reaction setup.

Single-factor experiments were conducted to optimize reaction conditions. Five temperature (35℃, 36℃, 37℃, 38℃, and 39℃) were tested with a fixed reaction time of 30 min. After determining the optimal temperature, six times points (5, 10, 15, 20, 25, 30 min) were evaluated. Amplification products were analyzed by agarose gel electrophoresis.

### Expression and purification of cas12a

The *Lachnospiraceae* bacterium ND2006 Cas12a (LbCas12a) was condon-optimized for expression in *E. coli* expression, and fused with a C-terminal 6×His tag. This gene fragment was cloned into the vector pET-28a (Beijing Bomaide Biotechnology Co., Ltd., China) to generate the recombinant plasmid pET28a-LbCas12a, which was then transformed into *E. coli* DH5α competent cells. A single positive colony was cultured in LB broth (Qingdao HopeBio Biotechnology Co., Ltd., China) supplemented with 50 µg/mL kanamycin at 37 °C and 220 rpm until OD_600_ reached 0.6 to 0.8. Expression was induced with 0.5 mM isopropyl-β-D-thiogalactopyranoside (IPTG) (Shanghai ShareBio Biotech Co., Ltd., China) at 16 °C and 180 rpm for 16 h. Cells were harvested by centrifugation at 8000 rpm for 10 min at 4 °C and resuspended in lysis buffer (20 mM Tris-HCl, 500 mM NaCl, 20 mM imidazole, pH 8.0). Cell lysis was performed by ultrasonication in an ice bath. After centrifuging at 12,000 rpm for 30 min at 4 °C, the supernatant was filtered through a 0.22 μm membrane. The recombinant Cas12a protein was purified using a Ni-NTA affinity chromatography column and eluted with buffer containing 20 mM Tris-HCl, 500 mM NaCl, and 250 mM imidazole (pH 8.0). The protein was dialyzed against a storage buffer (20 mM Tris-HCl, 150 mM NaCl, 1 mM DTT, 10% glycerol, pH 8.0) to remove imidazole. Protein concentration was measured using a BCA assay kit (Shanghai Beyotime Biotechnology Co., Ltd., China), and purity and integrity were confirmed by 10% SDS-PAGE (Shanghai Yamay Biomedical Technology Co., Ltd., China). Purified protein was aliquoted and stored at -80 °C for future CRISPR-Cas12a cleavage assays.

### Development and optimization of the CRISPR/Cas12a reaction system

CRISPR/Cas12a forms a ribonucleoprotein complex (RNP) with crRNA, recognizes the TTTN PAM site on the target DNA, and triggers cis- and trans-cleavage activities to non-specifically degrade single-stranded DNA. The conserved region of the *kmt1* gene was selected as the crRNA target. Conserved PAM sites with the TTTN motif were identified using SnapGene (v7.1.0). The 20 bp sequence downstream of these sites was fused with the crRNA conserved scaffold to design five candidate crRNAs (Table 2). Based on the trans-cleavage activity of cas12a, a fluorescent reporter probe with the sequence 5’-FAM-TTATT-Biotin-3’ (FB-ssDNA) was synthesized. Both crRNAs and probes were synthesized by Sangon Biotech (Shanghai, China). CrRNAs were dissolved in nuclease-free water to 1 µmol/L, aliquoted, and stored at -80 °C. To determine the optimal signal-to-noise ratio, a concentration gradient of the reporter probe (200, 400, 600, 800, and 1000 nmol/L) was systematically evaluated.

**Table 2:** crRNA sequence

Name	Sequences(5’→3’)
*kmt1*-crRNA1	UAAUUUCUACUAAGUGUAGAU*CCACACGCCAAAUAAAGACU*
*kmt1*-crRNA2	UAAUUUCUACUAAGUGUAGAU*GCGUGUGGCAAAGAAAAGCA*
*kmt1*-crRNA3	UAAUUUCUACUAAGUGUAGAU*UUUGCCACACGCCAAAUAAA*
*kmt1*-crRNA4	UAAUUUCUACUAAGUGUAGAU*TCGTGTATTGGTTACCTATTTTG*
*kmt1*-crRNA5	UAAUUUCUACUAAGUGUAGAU*GTGTGTTGAGCCAATCTGCTTCC*

crRNA consists of a DR sequence and a spacer sequence, with the underlined region representing the spacer sequence

The CRISPR/Cas12a reaction was carried out in a 20 µL containing 2 µL of 10× Cas12a buffer, 1 µL of Cas12a protein, 1 µL of crRNA, 2 µL of RPA amplification product, and 1 µL of the ssDNA reporter probe, followed by incubation at 37 °C for 30 min. Single-factor experiments were performed to optimize the concentrations of crRNA, Cas12a protein, and reporter probe, with cleavage efficiency and specificity as evaluation criteria. Five concentration levels (200, 400, 600, 800, and 1000 nmol/L) were tested separately while keeping all other conditions constant.

### Assembly of the lateral flow strip

On the nitrocellulose (NC) membrane of the colloidal gold nucleic acid test strip, the test line (T line) was coated with anti-FAM monoclonal antibody, while the control line (C line) was coated with an anti-gold-labeled mouse antibody. The gold-labeled probe was colloidal gold-conjugated streptavidin, which selectively binds to biotin moiety at the 3’ end of the reporter probe.

In the presence of the target sequence, Cas12a trans-cleavage activity is activated, cleaving the ssDNA reporter probe into short fragments labeled with FAM and biotin and dissociating the two functional groups. In positive samples, the free biotin-gold complex cannot be captured by the T line, resulting in a red signal only at the C line. Conversely, in negative samples, the intact FAM-biotin reporter probe forms a complex with the gold-labeled streptavidin, which is captured by both T and C lines, leading to dual-line color development. No color at the C line indicates an invalid strip.

### Sensitivity verification

To evaluate the sensitivity and limit of detection (LOD) of the assay, positive plasmid constructs containing the target sequence of the *P. multocida* reference strain were 10-fold serially diluted and thoroughly homogenized, yielding a continuous concentration gradient from 4.96 × 10¹⁰ to 4.96 × 10¹ copies/µL. The optimized RPA-CRISPR/Cas12a assay was performed using these diluted samples as templates. To ensure reproducibility, each concentration gradient was tested in triplicate (*n* = 3) during LOD determination, and the results were compared with those of conventional PCR.

The PCR reaction mixture (total 25 µL) contained 12.5 µL of 2× Taq Master Mix (Hunan Accurate Biological Engineering Co., Ltd., China), 1 µL of forward primer (10 µM), 1 µL of reverse primer (10 µM), 2 µL of extracted genomic DNA, and 8.5 µL of nuclease-free water. The specific primer sequences targeting the *kmt1* gene are listed in Table 3. The thermocycling parameters were: initial denaturation at 95℃ for 5 min; 35 cycles of 95℃ for 30 s, 48℃ for 30 s, and 72 °C for 45 s; with a final extension at 72℃ for 5 min. Amplified PCR products (450 bp) were analyzed by 1.5% agarose gel electrophoresis.

**Table 3:** Primer sequences for PCR amplification

Name	Sequences(5’→3’)	Length
PCR-F1	ATCCGCTATTTACCCAG	450 bp
PCR-R1	ACGAACTCGCCACTTTT	

### Specificity verification

Prior to wet-lab validation, an in silico alignment analysis was conducted via NCBI BLAST to evaluate the theoretical specificity of the designed primers and crRNA. The bioinformatics predictions confirmed that the sequences exhibited high identity exclusively with the target gene, showing significant mismatches against other non-target genomes. The assay specificity was evaluated against common respiratory pathogens coinfected with *P. multocida* and other prevalent pathogens, including *M. haemolytica*, *Histophilus somni*, bovine coronavirus, *Mycobacterium avium* subsp. *paratuberculosis*, bovine viral diarrhea virus, bovine parainfluenza virus type 3, bovine respiratory syncytial virus, bovine herpesvirus type 1, *E. coli*, and *Streptococcus species*. All the above strains were clinical isolates preserved in our laboratory. The species identity of each isolate was rigorously verified by species-specific PCR and Sanger sequencing.

### Repeatability verification

Three distinct concentrations of positive plasmids were selected, and each concentration was evaluated in triplicate technical replicates to assess intra-assay repeatability.

### Clinical sample detection

To assess the field applicability of the developed RPA-CRISPR/Cas12a-LFS assay, a blinded validation was conducted using 40 archived nasal swab specimens collected from large-scale commercial cattle farms in Shandong Province, China. All 40 specimens were derived from cattle exhibiting overt clinical signs of BRD, diagnosed by licensed veterinarians based on standard clinical scoring (e.g., body temperature > 39.5℃, purulent nasal discharge, persistent coughing, and dyspnea). No clinically healthy animals were included in this validation panel, and bacterial isolation and culture were not performed on these archived samples. Nasal swabs were selected because they provide a simple, non-invasive, and field-compatible sampling method that is well suited for POCT. Immediately after collection, swab heads were submerged in sterile PBS, transported under cold conditions, and stored at -80℃ until analysis to prevent repeated freeze-thaw cycles. Genomic DNA was extracted from the nasal swab suspensions using a commercial DNA Extraction Kit (Hunan Accurate Biological Engineering Co., Ltd., China) according to the manufacturer’s instructions. The diagnostic performance of the RPA-CRISPR/Cas12a-LFS assay was subsequently evaluated by comparison with conventional PCR, which served as the reference method.

## Results

### Assessment of the feasibility of the RPA-CRISPR/Cas12a-based detection technique

**Fig. 1:**
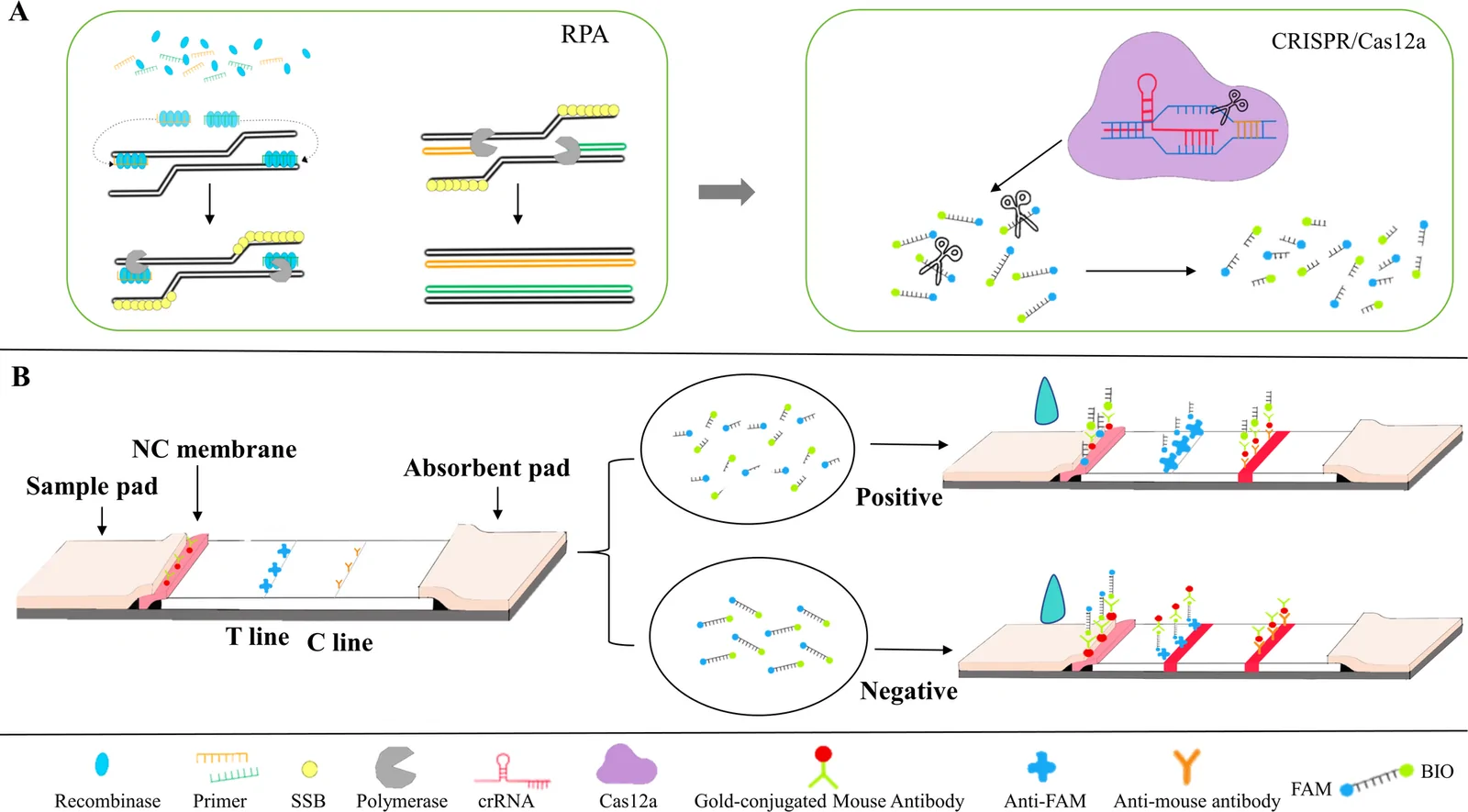
Schematic diagram of the working principle of the RPA-CRISPR/Cas12a visual detection system. **A** Schematic illustration of the coupled mechanism of RPA isothermal amplification and CRISPR/Cas12a reaction; the left part shows the RPA amplification process mediated by recombinase, primers and other components, and the right part shows the process of Cas12a recognizing the target and cleaving the reporter probe under the guidance of crRNA. **B** Schematic diagram of the detection principle and result interpretation of the LFS; the upper part shows the positive reaction (only C line appears colored), and the lower part shows the negative reaction (both T and C lines appear colored); the bottom part is the legend of each reaction component

A visual detection method integrating LFS technology was established by robust amplification capability of RPA and the trans-cleavage activity of the CRISPR/Cas12a. First, the target nucleic acid sequence was amplified and enriched by RPA. Then, CRISPR RNA (crRNA) and Cas12a enzyme were incubated at room temperature to form the Cas12a-crRNA complex. Addition of the RPA amplicon activated Cas12a trans-cleavage, leading to FB-ssDNA reporter probe and signal amplification. Finally, the reaction mixture was loaded onto the LFS, and the results were visually read by the naked eye after incubation at room temperature for 30 min (Fig. 1).

### Construction of recombinant plasmid standards

**Fig. 2:**
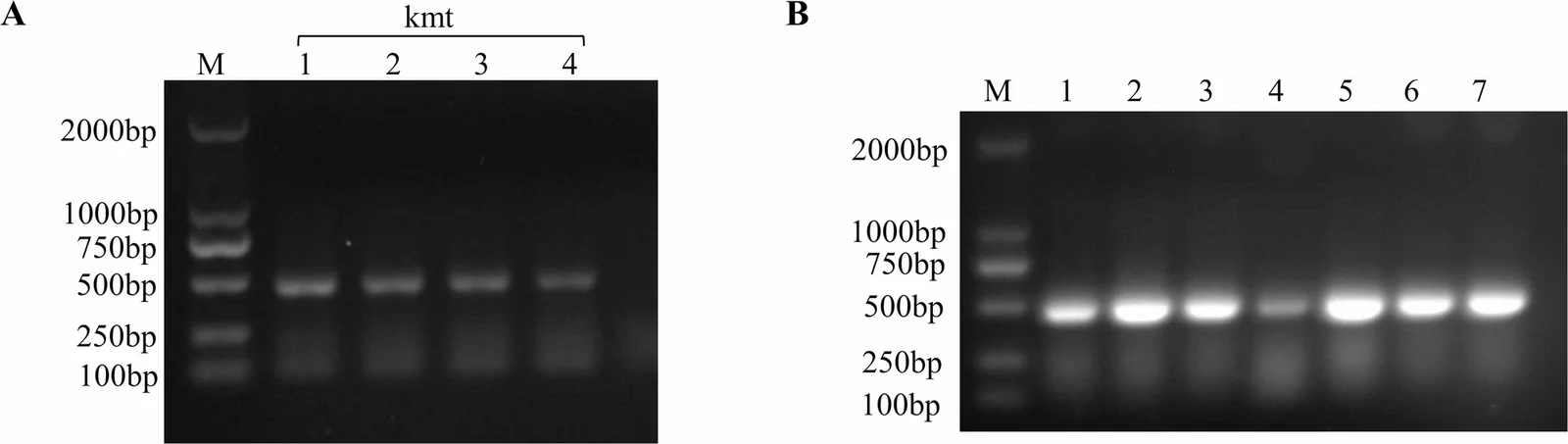
Construction of the recombinant plasmid pUC57EVL-*kmt1*. **A** Agarose gel electrophoresis analysis of PCR amplification products targeting the *kmt1* gene; M: DL2000 DNA marker; Lanes 1–4: PCR amplicons of the *kmt1* gene (496 bp). **B** Agarose gel electrophoresis of colony PCR for the identification of positive clones; M: DL2000 DNA marker; Lanes 1–7: PCR products derived from randomly selected single bacterial colonies

**Fig. 3:**
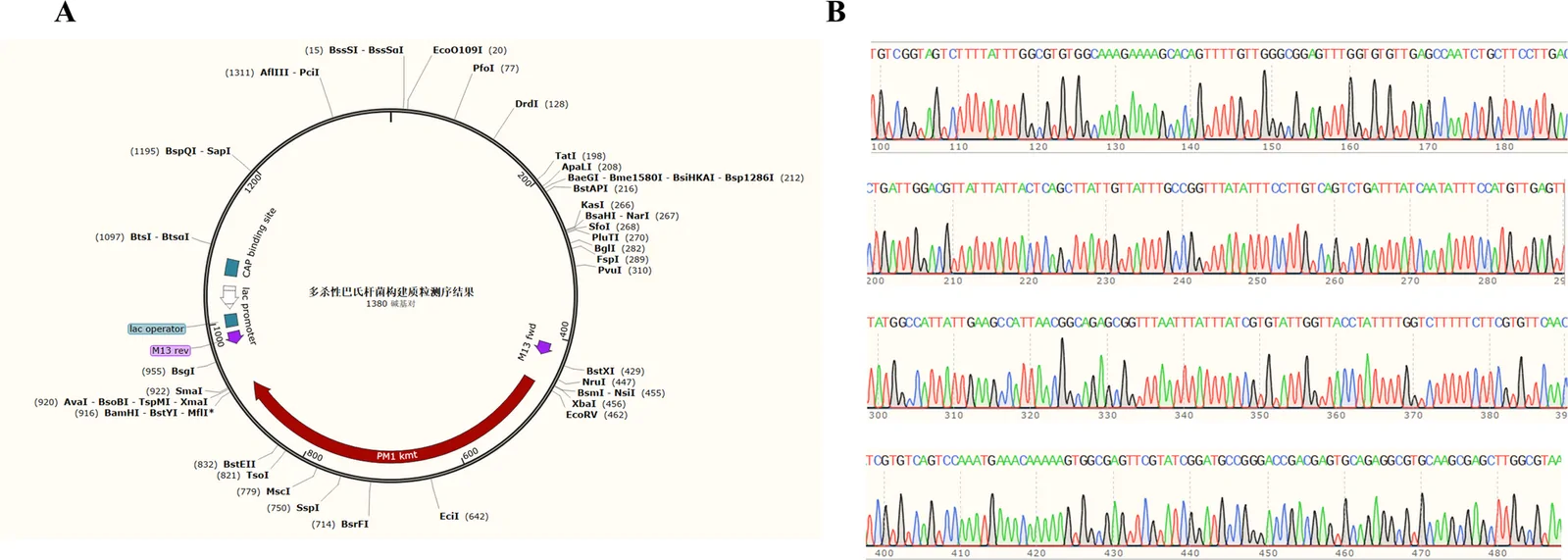
Construction and sequencing validation of the *P. multocida kmt1* gene-positive plasmid standard. **A** Restriction endonuclease site map of the recombinant plasmid carrying the *kmt1* gene; PM *kmt1* indicates the inserted target fragment. **B** Sanger sequencing chromatogram of the recombinant plasmid, confirming that the sequence of the inserted target fragment is consistent with the expected sequence

The *kmt1* gene was amplified by PCR using genomic DNA from the standard *P. multocida* strain. Agarose gel electrophoresis (1.5%) showed a single, distinct band at approximately 496 bp (Fig. 2A). The purified PCR product was seamlessly cloned into the EcoRV-linearized pUC57EVL vector and transformed into *E. coli* DH5α competent cells. Single colonies were successfully obtained on LB agar plates containing 100 µg/mL ampicillin. Five colonies were randomly selected for PCR verification and Sanger sequencing. Sequencing results confirmed that the inserted sequences were completely identical to the reference sequence (GenBank accession number KX348143.1) with no mismatches, insertions, or deletions, verifying the successful construction of the recombinant plasmid pUC57EVL-*kmt1* (Fig. 3).

The positive recombinant plasmid was extracted and quantified at 74.85 ng/µL using a NanoDrop 2000 spectrophotometer, corresponding to 4.96 × 10¹⁰ copies/µL. The plasmid was serial diluted 10-fold to prepare standard solutions ranging from 10⁷ to 10⁰ copies/µL, which were aliquoted and stored at -80 °C for future validation of methodological performance.

### Optimization of RPA system

**Fig. 4:**
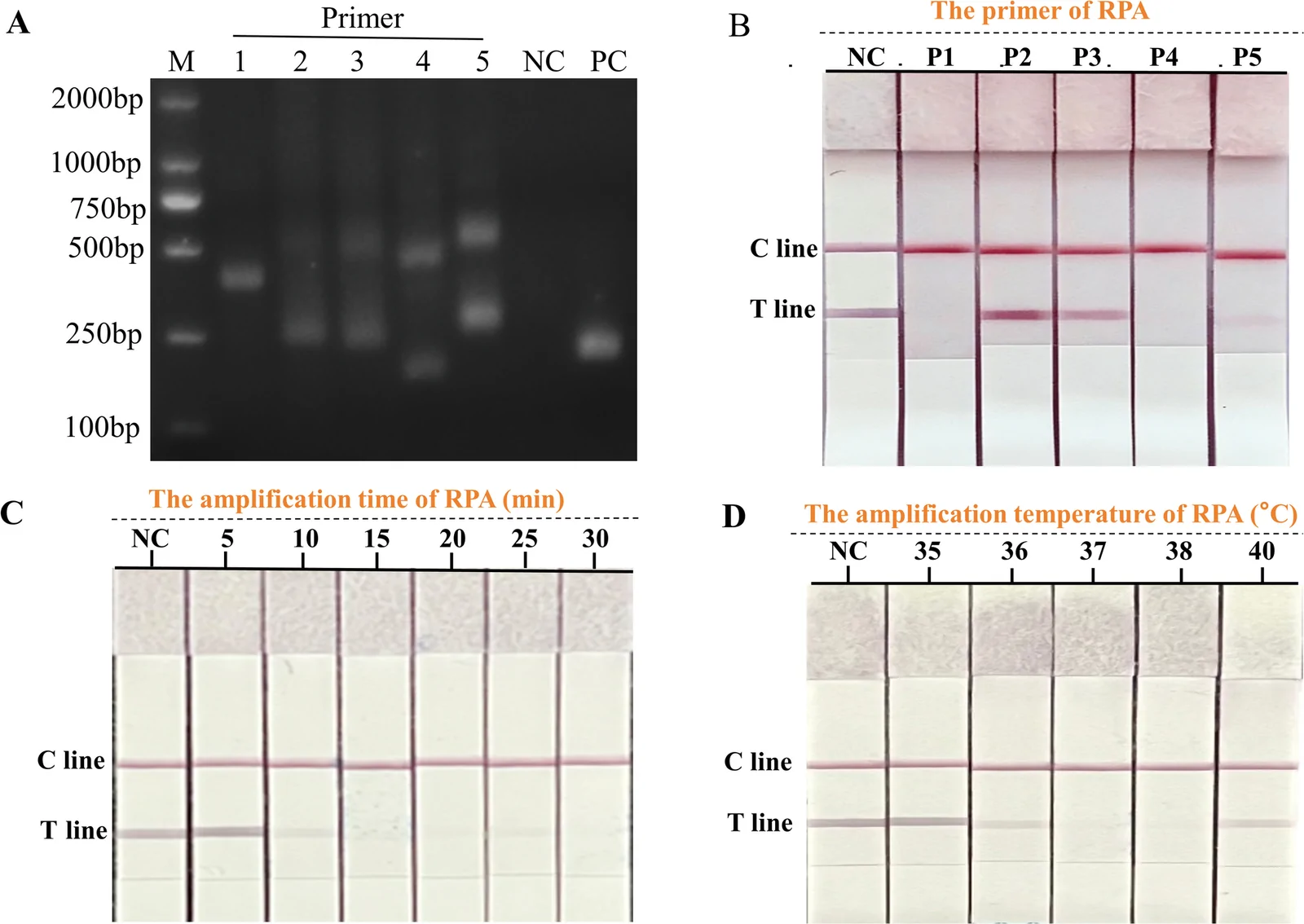
Screening of RPA primers and optimization of reaction conditions. **A** Agarose gel electrophoresis analysis of amplification products generated by five distinct pairs of RPA primers; M: DL2000 DNA marker; Lanes 1-5 correspond to primer pairs 1 through 5, respectively; NC: Negative control; PC: Positive control. **B** Lateral flow strip (LFS) detection outcomes for the five RPA primer pairs, designated P1 through P5. **C** Optimization of the RPA amplification duration, evaluated over a time span of 5 to 30 minutes. **D** Optimization of the RPA amplification temperature within the range of 35 to 40 °C. Images shown are representative of three independent experiments with similar results

Results of Optimal RPA Primer Screening. Electrophoretic analysis of amplification products generated by five distinct pairs of RPA primers (Fig. 4A, B) revealed that only primer pair 1 yielded a single, high-intensity target band without any non-specific amplification. In contrast, the remaining four primer pairs demonstrated either non-specific amplification or produced weak bands, suggesting suboptimal specificity and amplification efficiency. Consequently, primer pair 1 was identified as the most suitable candidate and was selected for use in subsequent experimental procedures.

Evaluation of Optimal Reaction Time for RPA Assay. The amplification outcomes across varying reaction time intervals revealed an absence of a discernible target band at 5 and 10 min. A distinct target band became evident at 15 min, with no notable variation in band intensity observed between 15 and 30 min. Consequently, to enhance detection efficiency, a reaction time of 15 min was established as optimal (Fig. 4C).

Optimization of the RPA Reaction Temperature: Screening Outcomes. The amplification outcomes across a range of temperature gradients indicated that the target band exhibited low intensity at 35 ℃ and 36 ℃. A distinct and well-defined target band was observed at 37 ℃, while no significant enhancement in band intensity was detected at 38 ℃ and 39 ℃. Consequently, 37 ℃ was identified as the optimal temperature for the reaction (Fig. 4D).

### Optimization of the CRISPR/Cas12a reaction system

**Fig. 5:**
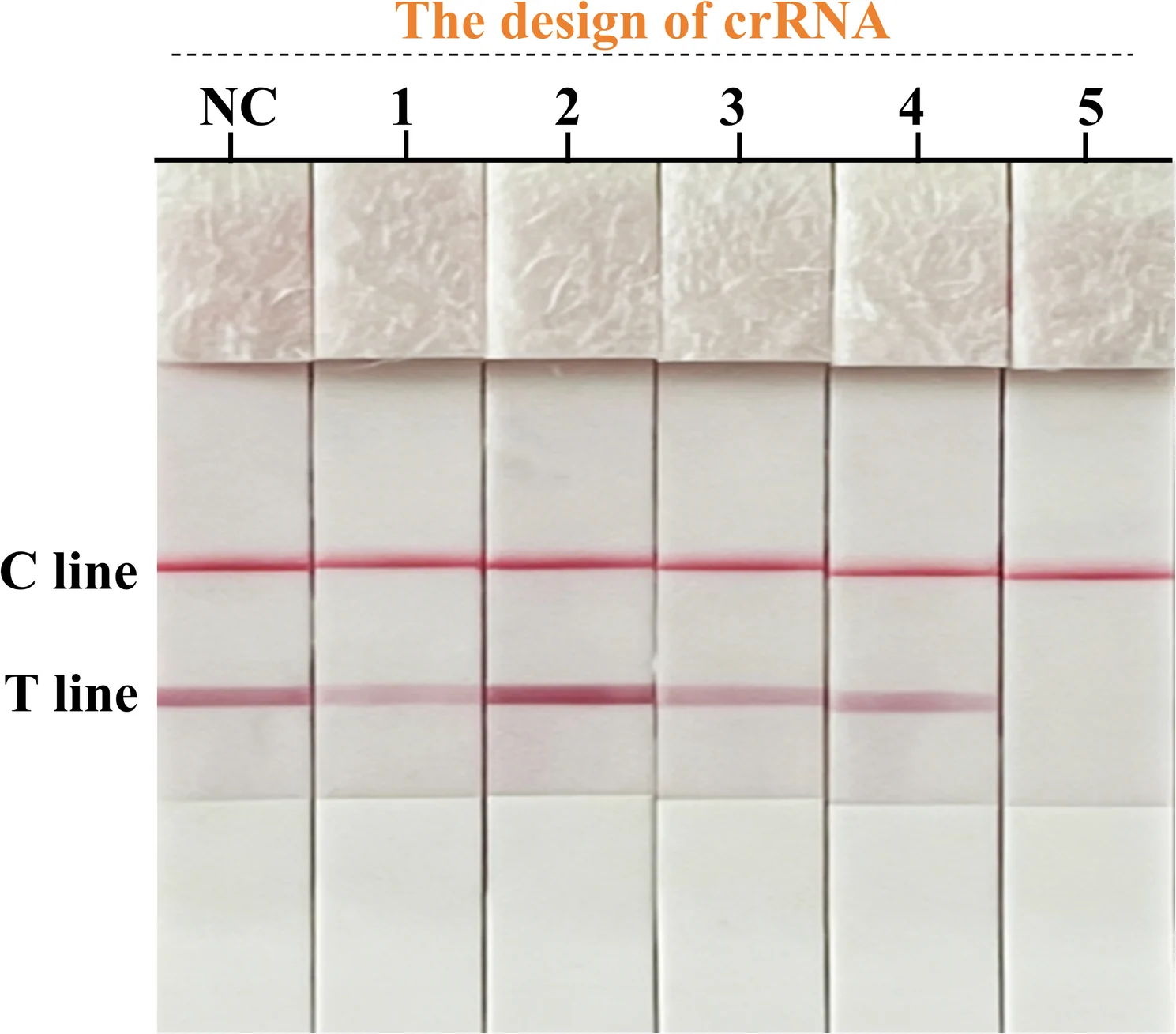
Results of LFS Screening for crRNA. NC: Negative control; 1–5: five candidate crRNAs; the absence of coloration on the T line indicates a positive result

Results of Optimal crRNA Screening. The colorimetric analysis of test strips for five distinct crRNAs (Fig. 5) revealed that the strip associated with crRNA-5 displayed a distinct control line (C line) without any visible test line (T line) signal. This pattern indicates a positive outcome characterized by the absence of false positives or background interference. In contrast, the other crRNAs demonstrated either faint T line coloration or yielded false-negative results. Consequently, crRNA-5 was identified as the optimal candidate for further application.

**Fig. 6:**
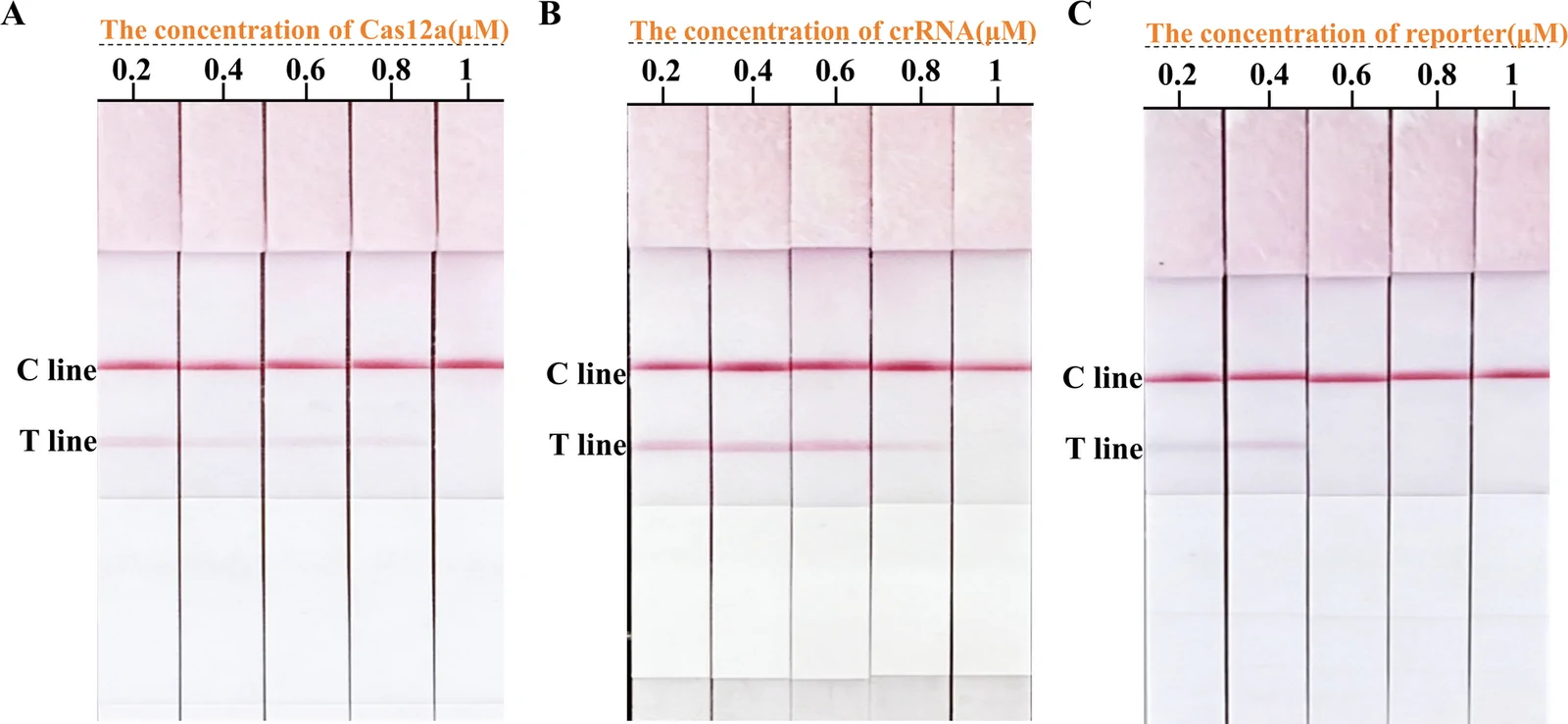
Optimization of component concentrations in the CRISPR/Cas12a reaction system. **A** Gradient of Cas12a protein concentrations ranging from 0.2 to 1 µM. **B** Gradient of crRNA concentrations ranging from 0.2 to 1 µM. **C** Gradient of FB-ssDNA reporter probe concentrations ranging from 0.2 to 1 µM. The absence of color development on the T line signifies a positive detection outcome. Images shown are representative of three independent experiments with similar results

Results of Optimal Reaction Concentration Screening. Optimization of the concentration gradient revealed that the test strip demonstrated the most distinct colorimetric response and highest specificity, with an absence of both false positive and false negative results, when the concentrations of crRNA, Cas12a protein, and ssDNA reporter probe were set at 1000 nmol/L, 1000 nmol/L, and 600 nmol/L, respectively. These values were identified as the optimal working concentrations for the three components, as illustrated in Fig. 6A, B, and C. Images shown are representative of three independent experiments with similar results.

### Sensitivity analysis

**Fig. 7:**
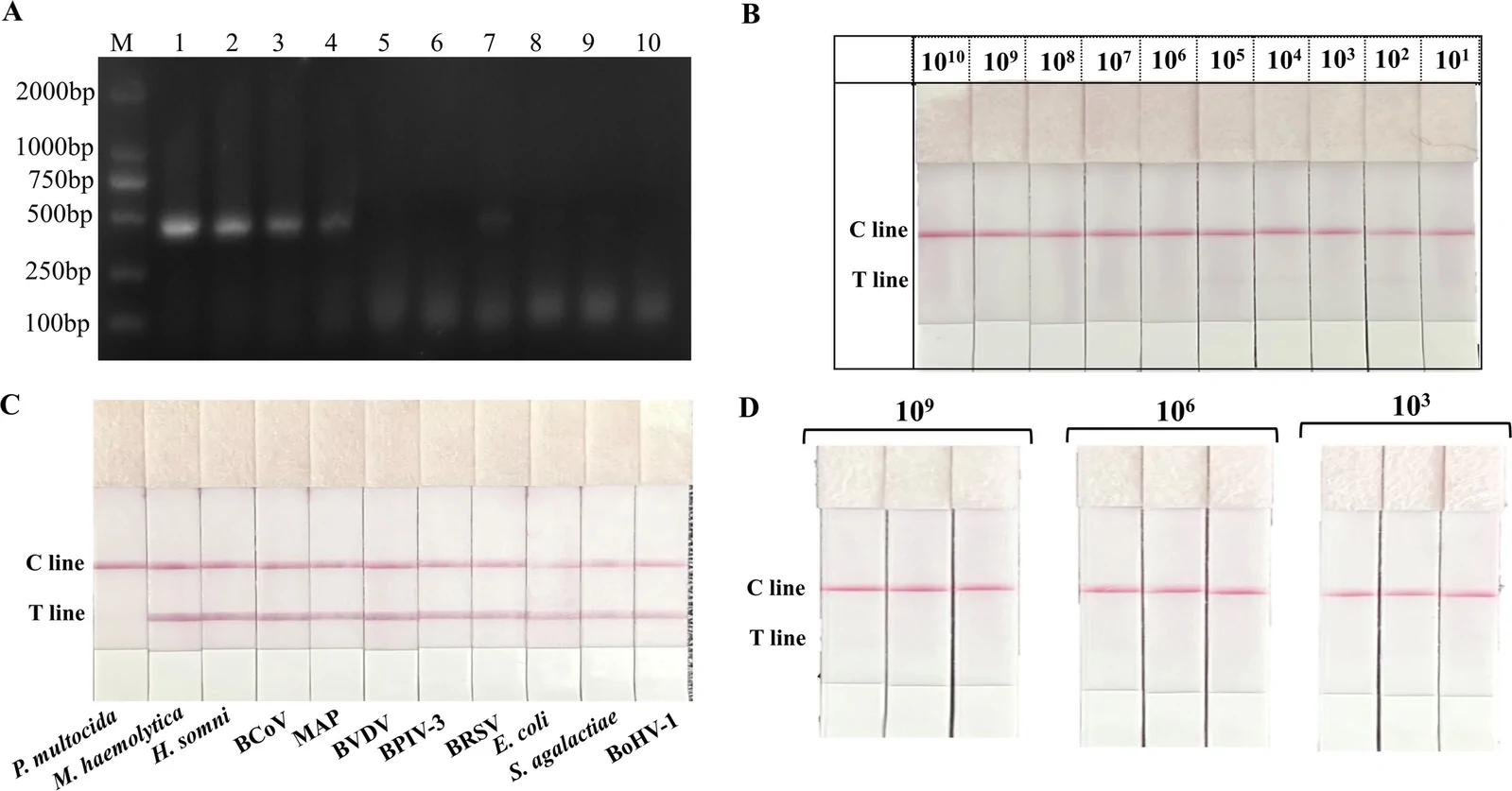
Verification of sensitivity, specificity and repeatability of the RPA-CRISPR/Cas12a-LFS assay. **A** Electrophoretogram of conventional PCR sensitivity detection; M: DL2000 DNA Marker; Lanes 1–10: Plasmid standards at 10¹⁰-10¹ copies/µL. **B** Sensitivity detection results of RPA-CRISPR/Cas12a-LFS; Lanes 1–10: Plasmid standards at 10¹⁰-10¹ copies/µL. **C** Specificity detection results; From left to right: *Pasteurella multocida*, *Mannheimia haemolytica*, *Histophilus somni*, bovine coronavirus, *Mycobacterium avium* subsp. *paratuberculosis*, bovine viral diarrhea virus, bovine parainfluenza virus type 3, bovine respiratory syncytial virus, *Escherichia coli*, *Streptococcus agalactiae*, bovine herpesvirus type 1. **D** Repeatability detection results; From left to right: Three parallel replicates of samples at concentrations of 10⁹, 10⁶ and 10³ copies/µL. No color development on the T line indicates a positive result. Images shown are representative of three independent experiments with similar results

The detection results of serial concentrations of positive plasmids showed that the limit of detection (LOD) of the established RPA‑CRISPR/Cas12a assay was 10¹ copies/µL, at which clear positive results could still be observed on the test strip. In contrast, the limit of detection of conventional PCR was 10⁷ copies/µL, indicating that the sensitivity of the RPA‑CRISPR/Cas12a method was 10⁶‑fold higher than that of conventional PCR, demonstrating its high‑sensitivity detection capability (Fig. 7A, B).

### Specificity analysis

The specificity test results demonstrated that distinct positive reactions, characterized by a clear C line and the absence of color development on the T line, were exclusively observed in the *P. multocida* positive control and positive plasmid samples. Ten control pathogens, including bovine viral diarrhea virus, bovine respiratory syncytial virus, as well as the negative control, all exhibited double bands indicative of negative results, with no evidence of cross-reactivity (Fig. 7C). These findings indicate that the developed method exhibits high specificity.

### Repeatability analysis

In the intra-assay repeatability experiment, three parallel replicates of samples at high, medium, and low concentrations all showed consistent color development results without variation (Fig. 7D). These results indicate that the method has good repeatability and provides stable and reliable results.

### Clinical sample analysis

**Fig. 8:**
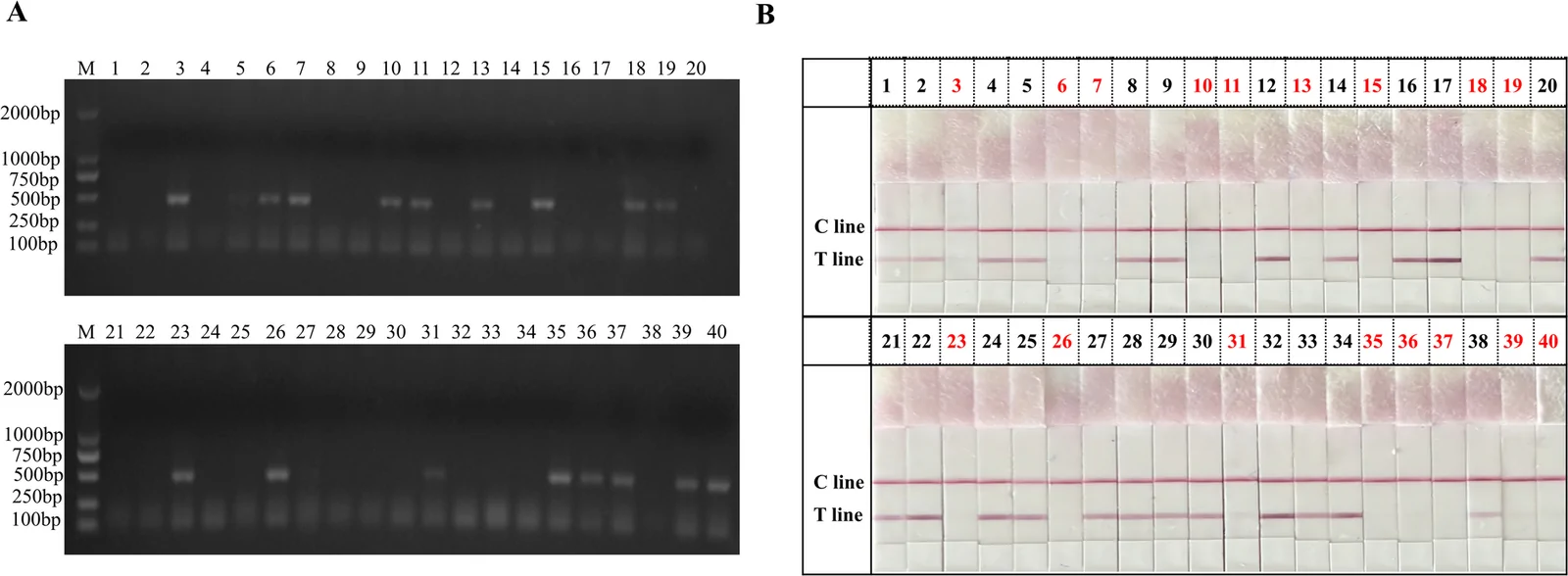
Detection outcomes for clinical specimens. **A** Electrophoretic analysis of conventional PCR assays conducted on 40 clinical nasal swab samples; M denotes the DL2000 DNA marker; lanes 1 through 40 correspond to individual clinical samples. **B** Results of RPA-CRISPR/Cas12a-LFS assays performed on the same clinical samples; samples yielding positive results are indicated by red numerals. The absence of color development on the test (T) line signifies a positive detection. Images shown are representative of three independent experiments with similar results

A total of 40 clinical samples were analyzed in parallel using both assays. Conventional PCR identified 17 positive and 23 negative samples, and the RPA-CRISPR/Cas12a assay yielded identical results, also detecting 17 positive and 23 negative samples. (Fig. 8A, B). Detailed comparative data are summarized in Table 4. The RPA-CRISPR/Cas12a assay showed 100% sensitivity and 100% specificity compared with conventional PCR, indicating a 100% diagnostic agreement between the two methods for clinical specimen screening.

To further evaluate the quantitative performance and detection capacity across varying pathogen loads, an additional cohort of 42 clinical nasal swab specimens was analyzed using real-time qPCR as the standard reference method (Supplementary Table S1). The reference qPCR Ct values ranged from 16.0 to 35.0, representing a broad spectrum of bacterial abundances. The RPA-CRISPR/Cas12a-LFS assay exhibited 100% agreement with qPCR (36 positives and 6 negatives), reliably detecting low-abundance specimens even at a high Ct value of 35.0.

**Table 4:** Agreement between RPA/Cas12a assay and PCR for detection of *Pasteurella multocida* in clinical samples

Method	Result	PCR	
		Positive	Negative
RPA/Cas12a	Positive	17	0
	Negative	0	23

## Discussion

*P. multocida* is a major bacterial pathogen associated with BRD, causing considerable economic losses in cattle production worldwide [20, 21]. Rapid and accurate detection of *P. multocida* is essential for timely disease control and epidemiological surveillance, particularly in field and resource-limited veterinary settings. However, conventional bacterial isolation and culture procedures are labor-intensive and time-consuming, while PCR-based methods generally require specialized laboratory equipment and trained personnel. Therefore, the development of rapid, sensitive, and field-deployable diagnostic methods remains critical for improving the management of bovine respiratory disease.

In the present study, an LFS-based RPA-CRISPR/Cas12a assay targeting the species-specific *kmt1* gene of *P. multocida* was successfully developed for rapid visual detection. RPA technology has been increasingly applied in point-of-care molecular diagnostics because of its rapid amplification efficiency and low equipment requirements [10]. Compared with conventional PCR, RPA amplification can be performed under constant low-temperature conditions without the need for thermal cycling equipment, which improves its suitability for field applications [9, 22]. In addition, compared with loop-mediated isothermal amplification (LAMP), RPA generally requires a shorter reaction time and simpler primer design, making it more convenient for rapid pathogen detection in veterinary practice [23]. While post-amplification tube opening poses a recognized risk of aerosol carry-over contamination in isothermal platforms, our strategy minimizes this limitation through two complementary mechanisms [24]. First, the pre-treatment and incorporation of the UDG-dUTP system enzymatically eliminates residual DNA contaminants. Second, the integration of the CRISPR/Cas12a detection system provides a high-stringency sequence recognition barrier [25]. Because Cas12a cleavage requires strict crRNA-target complementarity, minor aerosol artifacts or non-specific amplicons fail to trigger reporter cleavage [11]. This dual-protection architecture ensures maximum accuracy and zero false-positive rate, facilitating robust decentralized POCT.

Recently, CRISPR/Cas12a-based detection systems have attracted increasing attention because of their high specificity and signal amplification capability [26, 27]. Guided by a specific CRISPR RNA (crRNA), the Cas12a endonuclease precisely recognizes target DNA sequences and subsequently triggers the trans-cleavage of single-stranded DNA (ssDNA) reporters, enabling visual or fluorescent signal generation [26, 28]. Previous studies have reported fluorescence-based RPA-CRISPR/Cas12a assays for the detection of *P. multocida*. Although these methods demonstrated good analytical performance, fluorescence-based signal detection still depends on specialized fluorescence readers or laboratory instruments, limiting their practical applicability in resource-limited settings [18, 29]. In contrast, the lateral flow strip (LFS)-based visualization used in the present study enables direct visual interpretation without complex instrumentation [30]. This feature improves the practicality and accessibility of the assay for POCT and field deployment in veterinary diagnostics.

The *kmt1* gene was selected as the detection target because it is highly conserved and species-specific for *P. multocida* [31]. No cross-reactivity was observed with other common bovine pathogens included in this study, indicating the excellent analytical specificity of the assay. Furthermore, repeatability testing demonstrated stable assay performance, supporting its reliability for routine diagnostic applications. It should be noted that *P. multocida* can act as an opportunistic pathogen or commensal organism in the upper respiratory tract of cattle. Consequently, mere DNA detection without clinical context may reflect colonization rather than active infection. However, in acute clinical presentations (such as acute pasteurellosis or hemorrhagic septicemia), bacterial loads increase substantially. The high sensitivity of this RPA-CRISPR/Cas12a assay is designed primarily for rapid on-site screening and early decision-making in field settings. When integrated with overt clinical signs, a rapid positive result provides actionable intelligence for immediate isolation and targeted therapeutic intervention. To further enhance clinical specificity, future iterations of this platform could incorporate semi-quantitative threshold controls to effectively distinguish between high-load acute infection and low-level commensal colonization.

Clinical sample testing demonstrated complete concordance between the LFS-based RPA-CRISPR/Cas12a assay and conventional PCR. Although the number of clinical samples included in this study was relatively limited, these preliminary results indicate the potential applicability of the assay for practical veterinary diagnosis. Additional validation using larger clinical sample cohorts from different geographical regions and production systems will be necessary to further evaluate the diagnostic performance of the assay under diverse field conditions. Furthermore, evaluating clinical performance against indexed qPCR Ct values is essential for POCT field applicability. Through real-time qPCR validation of clinical specimens, the reference Ct values for positive samples ranged from 16.0 to 35.0 (Supplementary Table S1). Notably, our RPA-CRISPR/Cas12a-LFS assay successfully detected all qPCR-positive samples, maintaining clear visual readouts even for low-abundance specimens near the qPCR detection threshold (Ct up to 35.0). This demonstrates the robust analytical sensitivity and reliability of our platform under varying pathogen loads.

Nevertheless, this study still has several limitations. First, the sample size for clinical validation was relatively small. Second, the assay’s robustness under variable environmental conditions (e.g., ambient temperature fluctuations and non-standard field operations) requires further systematic quantification. Third, the present assay was designed as a single-target detection method, whereas bovine respiratory disease is often associated with multiple pathogens. Future studies should focus on the development of multiplex CRISPR-based detection platforms capable of simultaneously detecting multiple respiratory pathogens, such as *M. haemolytica* and *M. bovis*. Fourth, DNA was extracted using a standard commercial kit. Rapid, field‑compatible extraction methods such as direct lysis buffers were not tested in this study. Assessing the compatibility of rapid lysis reagents with downstream RPA reactions is necessary for true point‑of‑care application. In addition, further optimization of reagent preservation and portable sample preparation procedures may further improve the field applicability of the assay.

## Conclusions

In conclusion, the present study aims to present a newly developed LFS-based RPA-CRISPR/Cas12a assay for the rapid, visual detection of *P. multocida*. The assay demonstrated high sensitivity, excellent specificity, and favorable repeatability, showing complete agreement with conventional PCR in clinical sample validation. Compared with fluorescence-based detection systems, this LFS-based platform offers a simpler, instrument-independent readout, rendering it highly suitable for POCT and field diagnosis in veterinary settings. This study provides a practical molecular diagnostic approach for the rapid surveillance of *P. multocida* and may contribute significantly to the improved control and management of bovine respiratory disease within cattle production systems.

## Supplementary Information

Supplementary Material 1.

## Data Availability

All data generated or analyzed during this study are included in this published article. The raw data supporting the conclusions of this study are available from the corresponding author upon reasonable request.
