# Supplementary material for: Rapid on-site detection of Pasteurella multocida in cattle using an LFS-based RPA-CRISPR/Cas12a assay
Source: Ir Vet J. 2026 Sep 7;79:38. doi: 10.1186/s13620-026-00360-4 (PMC13617714; doi:10.1186/s13620-026-00360-4)
Supplement: Supplementary file 1 [file 13620_2026_360_MOESM1_ESM.docx]

## Supplementary Materials

Manuscript ID: 04d2ef86-70d3-4d68-8585-84bc54b0c909

Title: Rapid on-site detection of *Pasteurella multocida* in cattle using an LFS-based RPA-CRISPR/Cas12a assay

**Section S1. Real-Time Quantitative PCR (qPCR) Assay**

To evaluate the diagnostic capability across varying pathogen loads (Ct values) and validate the performance of the developed RPA-CRISPR/Cas12a-LFS assay, a quantitative real-time PCR (qPCR) targeting the *kmt1* gene was performed on an additional cohort of 42 archived clinical bovine nasal swab specimens (DNA extracted).

**S1.1 Primers, Reagents, and Reaction Conditions**

The qPCR assay was conducted using SYBR Green qPCR Master Mix (Hunan Accurate Biological Engineering Co., Ltd., Changsha, China) on a real-time PCR detection system (Yuanzan Biotechnology Co., Ltd., China).

The specific primer sequences targeting the *kmt1* gene are listed in Supplementary Table S1. The thermocycling parameters consisted of initial denaturation at 95°C for 10 min, 40 cycles of 95°C for 5 s and 60°C for 30 s, followed by melting curve analysis (95°C for 10 s, 65°C for 1 min, and 98°C for 1 s).

**S1.2 Evaluation of Clinical Specimens**

A sample was defined as qPCR-positive if a typical logarithmic amplification curve was observed with a Ct value ≤35.0. Among the 42 clinical nasal swab DNA samples tested, 36 were identified as positive (Ct values ranged from 16.0 to 35.0) and 6 were identified as negative (Supplementary Table S2).

The LFS-based RPA-CRISPR/Cas12a assay was evaluated in parallel on these 42 samples. The results showed 100% diagnostic agreement with the reference qPCR assay (36 positive and 6 negative results), successfully detecting low-abundance specimens even at a high Ct value of 35.0.

**Table S1.** Primer sequences for qPCR amplification

| Name | Sequences（5'→3'） | Length |
| --- | --- | --- |
| qPCR-F1 | AGTGGGCTTGTCGGTAGTCT | 124bp |
| qPCR-R1 | AATAACGTCCAATCAGTTGC |  |

**Table S2.** Clinical Sample Detection Results

| Sample | Ct value | Sample | Ct value |
| --- | --- | --- | --- |
| Positive control | 16.32±0.03 | Negative control | 35.54±0.29 |
| 1 | 29.27±1.02 | 22 | 34.09±1.24 |
| 2 | 30.25±0.33 | 23 | 34.20±1.35 |
| 3 | 30.40±0.62 | 24 | 33.85±1.58 |
| 4 | 30.46±0.30 | 25 | 33.40±0.93 |
| 5 | 29.21±0.25 | 26 | 34.43±1.28 |
| 6 | 24.08±0.13 | 27 | 33.00±0.13 |
| 7 | 27.69±0.14 | 28 | 32.52±0.10 |
| 8 | 28.09±6.80 | 29 | 32.77±0.71 |
| 9 | 27.82±0.08 | 30 | 33.00±0.59 |
| 10 | 28.59±0.38 | 31 | 34.09±1.23 |
| 11 | 17.50±0.04 | 32 | 34.86±0.92 |
| 12 | 27.80±0.06 | 33 | 34.44±0.49 |
| 13 | 28.79±0.06 | 34 | 34.94±0.70 |
| 14 | 25.89±0.07 | 35 | 34.84±0.12 |
| 15 | 31.08±0.37 | 36 | 34.79±0.13 |
| 16 | 33.54±0.37 | 37 | 36.29±0.29 |
| 17 | 33.65±0.78 | 38 | 35.66±0.48 |
| 18 | 33.89±0.73 | 39 | 35.61±0.49 |
| 19 | 34.06±0.71 | 40 | 39.55±0.38 |
| 20 | 34.07±0.23 | 41 | 35.86±1.39 |
| 21 | 34.76±1.01 | 42 | 36.40±1.17 |

**Figure 1S.** Visual detection results of 42 clinical bovine nasal swab specimens using the RPA-CRISPR/Cas12a-LFS assay

Numbers 1 to 42 represent the individual archived clinical nasal swab samples (corresponding to Sample IDs 1 to 42 in Supplementary Table S1). Lanes 1–36 represent qPCR-positive clinical specimens (Ct values ranging from 16.0 to 35.0), while Lanes 37–42 represent qPCR-negative specimens. The presence of both the control line (C line) and the test line (T line) indicates a positive result, whereas the presence of only the C line indicates a negative result. The assay achieved 100% concordance with reference qPCR results across all 42 clinical samples.

**Table S3.** Primer sequences for pUC57EVL-PCR amplification

| Name | Sequences（5'→3'） | Length |
| --- | --- | --- |
| pUC57-kmt1-F | CCTCGCGAATGCATCTAGATATCCGCTATTTACCCAG | 490bp |
| pUC57-kmt1-R | CGACGGGCCCGGGATCCGATACGAACTCGCCACTTTT |  |

**Section S2. Identification and Re‑verification of Strains**

**Table S4.** Strain Information and Primer Sequences

| Name | Target gene | Primer | length | GenBank |
| --- | --- | --- | --- | --- |
| *P. multocida* | *kmt1* | F: ATCCGCTATTTACCCAG  R: ACGAACTCGCCACTTTT | 450bp | KX348143.1 |
| *M. haemolytica* | gcp | F: TTTCCGTTTGTGGCATTAT  R: TATTCGCAGCAAAGGTTTT | 280bp | AY839677.1 |
| *H. somni* | 16S rRNA | F: CCTGGTAGTCCACGCTGTA  R: GCTCGCCTCCCTCTGTAT | 466bp | PQ533026.1 |
| BCoV | N | F: GCTATTCCGACTAGGTTTC  R: CTTAGTTACTTGCTGTGGC | 258bp | NP_150083.1 |
| MAP | IS900 | F: CGTCGGGAGTTTGGTAGCC  R: ACGTTCCGATCTGGTGGC | 149bp | S74401.1 |
| BVDV | 5‘UTR | F: CCGCGAAGGCCGAAAAGA  R: TGACGACTNCCCTGTACTCAG | 116bp | AF091605.1 |
| BPIV-3 | M | F: GAAAGCGACCGAAATGAT  R: GCGGTGCAGTTCACAAAA | 177bp | KC011288.1 |
| BRSV | N | F: TGCTATGTGTTGCTGCTTTGGTTA  R: TCTTGATTGCCTCTAATTCCTCTGTAGT | 660bp | NC_001989.1 |
| *E. coli* | uidA | F: ACTGGCTTTGGTCGTCAT  R: CTCTTCAGCGTAAGGGTAAT | 138bp | S69414.1 |
| *S. agalactiae* | cfb | F: CCCAGCAAATGGCTCAAA  R: AAGGCTTCTACACGACTACCAA | 193bp | X72754.1 |
| BoHV-1 | gB | F: GCACCGCCAACCACAAG  R: CCCAGGCCCTGAAAGAAG | 331bp | AJ004801.1 |

**S2. Molecular identification and sequence verification of clinical bacterial isolates.**

**Figure S2.** Representative 1.5% agarose gel electrophoresis of species-specific PCR products for target and non-target bacterial strains. Lane M: DNA ladder (100–2000 bp); Lane 1: *P. multocida* (target gene *kmt1*); Lanes 2–11: Non-target control pathogens (*Mannheimia haemolytica*, *Histophilus somni*, bovine coronavirus, *Mycobacterium avium* subsp*. paratuberculosis*, bovine viral diarrhea virus, bovine parainfluenza virus type 3, bovine respiratory syncytial virus, *Escherichia coli*, *Streptococcus agalactiae*, bovine herpesvirus type 1).

**Figure S3.** Sanger sequencing result and alignment of *P. multocida*.

Representative Sanger sequencing chromatogram and NCBI BLAST alignment of *P. multocida*, showing high-quality single peaks and 100% sequence identity to the reference genome.

**Figure S4.** Sanger sequencing result and alignment of *M. haemolytica*.

Representative Sanger sequencing chromatogram and NCBI BLAST alignment of *M. haemolytica*, showing high-quality single peaks and 100% sequence identity to the reference genome.

**Figure S5.** Sanger sequencing result and alignment of *H. somni*.

Representative Sanger sequencing chromatogram and NCBI BLAST alignment of *H. somni*, showing high-quality single peaks and 100% sequence identity to the reference genome.

**Figure S6.** Sanger sequencing result and alignment of BCoV.

Representative Sanger sequencing chromatogram and NCBI BLAST alignment of BCoV, showing high-quality single peaks and 100% sequence identity to the reference genome.

**Figure S7.** Sanger sequencing result and alignment of MAP.

Representative Sanger sequencing chromatogram and NCBI BLAST alignment of MAP, showing high-quality single peaks and 100% sequence identity to the reference genome.

**Figure S8.** Sanger sequencing result and alignment of BVDV.

Representative Sanger sequencing chromatogram and NCBI BLAST alignment of BVDV, showing high-quality single peaks and 100% sequence identity to the reference genome.

**Figure S9.** Sanger sequencing result and alignment of BPIV-3.

Representative Sanger sequencing chromatogram and NCBI BLAST alignment of BPIV-3, showing high-quality single peaks and 100% sequence identity to the reference genome.

**Figure S10.** Sanger sequencing result and alignment of BRSV.

Representative Sanger sequencing chromatogram and NCBI BLAST alignment of BRSV, showing high-quality single peaks and 100% sequence identity to the reference genome.

**Figure S11.** Sanger sequencing result and alignment of *E. coli*.

Representative Sanger sequencing chromatogram and NCBI BLAST alignment of *E. coli*, showing high-quality single peaks and 100% sequence identity to the reference genome.

**Figure S12.** Sanger sequencing result and alignment of *S. agalactiae*.

Representative Sanger sequencing chromatogram and NCBI BLAST alignment of *S. agalactiae*, showing high-quality single peaks and 100% sequence identity to the reference genome.

**Figure S13.** Sanger sequencing result and alignment of BoHV-1.

Representative Sanger sequencing chromatogram and NCBI BLAST alignment of BoHV-1, showing high-quality single peaks and 100% sequence identity to the reference genome.
